# Oral manifestations of systemic amyloidosis, an aid to diagnosis of multiple myeloma – report of two cases

**DOI:** 10.1016/j.bjorl.2020.11.011

**Published:** 2020-12-19

**Authors:** D.W.V.N. Dissanayaka, H.M.M.R. Bandara, T. Sabesan, Y.S. Mohomed, B.S.M.S. Siriwardena

**Affiliations:** a University of Peradeniya, Faculty of Dental Sciences, Department of Oral Pathology, Kandy, Sri Lanka; b Ministry of Health, Cota, Sri Lanka

## Introduction

Amyloidosis is a systemic or localized accumulation of insoluble fibrillar proteins. Amyloid is a special type of extracellular amorphous eosinophilic proteinaceous deposit with distinctive tinctorial characteristics with congo red stain. It has a salmon pink color under ordinary light and shows a diagnostic apple green birefingence under polarized light. Oral manifestations of amyloidosis are well recognized. The most frequently reported location for intraoral amyloid deposition is the tongue; deposition on the tongue may result in macroglossia and firm tongue.^1^, ^2^ Commonly the deposition appeared as waxy papules or nodules on various oral surfaces including the tongue as well as buccal mucosa. However deposition of amyloid on the tongue is very rare and accounts for less than 9% of all types of amyloidosis.^2^

Amyloidosis often develops as a systemic disease which can be associated with multiple myeloma (MM). MM is a rare, largely incurable malignant disease characterized by uncontrolled proliferation of clonal plasma cells producing a monoclonal paraprotein, mainly IgG (55%) or IgA (20%) and rarely IgM and IgD causing a wide variety of complications leading to organ dysfunction and eventually death.^2^ The paraproteinemia may be associated with excretion of light chains in the urine (Bence Jones proteins). It is a disease of the elderly, the median age at presentation being over 60yrs, and rare under 40yrs. MM is suspected in patients presenting with back pain, bone pain (accompanied or not by pathological fractures), unexplained anemia, renal insufficiency and/or hypercalcemia, recurrent infections and secondary amyloidosis.^3^ Frequently, bone lesions may present with diffuse or localized osteolytic lesions, named plasmacytomas, or by a ‘punched-out’ pattern. Maxillary and mandibular bones may be affected by these lesions. Nearly 80% of diagnosed MM is preceded by asymptomatic a premalignant stage termed monoclonal gammopathy of undetermined significance (MGUS) and nearly 35% of patients diagnosed with symptomatic MM present lesions in the jaws.^4^ In this paper we report two cases of multiple myeloma in which oral manifestations were the primary indicators.

## Case report

### Case 1

**Figure 1:**
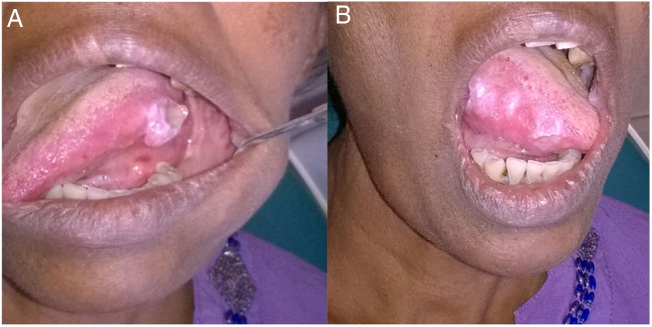
(A) Ulcerative lesions on lateral border of the tongue. (B) Multiple lesions on the other side of the tongue.

A 57-year-old female patient presented to District General Hospital, Negambo with multiple painful oral ulcerative lesions on tongue and bilateral buccal mucosa for 1 year and 6 months duration. She complained about marked weight loss and loss of appetite for a period of about 2 months and history of rectal bleeding, anal discomfort and constipation. Intraoral examination revealed ulcers on the lateral borders of the tongue (Fig. 1a and b) and small ulcers on the buccal mucosa.

Hematological investigations revealed low monocyte, red blood cells (RBC) and hemoglobin counts and low hematocrit. High levels of erythrocyte sedimentation rate (ESR) with high eosinophil counts were also revealed. Moreover, the blood picture was reported as anemia of chronic disease with or without iron deficiency anemia. An ultrasound scan of abdomen and pelvis revealed fatty liver without hepatosplenomegaly.

Incisional biopsy was performed from one of ulcers of the tongue and the corium was composed of a homogenous eosinophilic material suggesting amyloid. This was confirmed with congo red stain and typical apple-green birefringence under polarized light. General medical evaluation of the patient revealed renal dysfunction, anemia, hypercalcemia, and hyperphosphatemia. The pathologist suggested investigating the patient for multiple myeloma, which resulted in a referral to hematologists. Bone marrow core biopsy revealed plasma cell dyscrasia. Further biochemical and amyloid deposits were also observed in blood vessels. Finally the condition was diagnosed as amyloid light-chain (AL) amyloidosis. One month after the diagnosis patient passed away.

### Case 2

**Figure 2:**
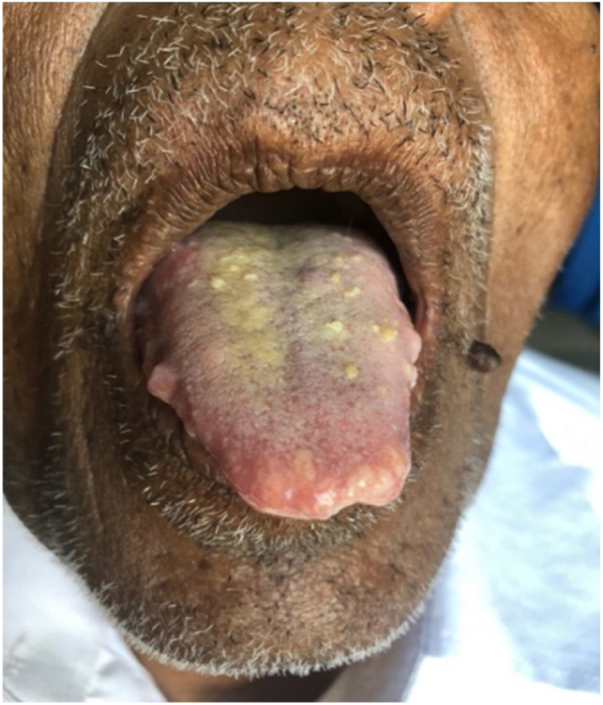
Yellowish small nodules on dorsal tongue.

A 76-year-old male patient with small yellowish nodules on mucosal surfaces of the tongue (Fig. 2), buccal mucosa and lips with the main complaint of difficulty in swallowing presented to General Hospital, Polonnaruwa. On examination there were yellowish papules about 0.5 × 0.5 cm in diameter and he complained that the tongue was sore and enlarged. The papules were soft in consistency. Multiple whitish plaques were also present on lateral borders of the tongue and were approximately 2 cm in diameter. The patient also had multiple nodules in the region of the buccal mucosa near the labial commissure of the right side, with approximately 1 cm each with pain, firm consistency and spontaneous bleeding. He was also suffered from severe constipation. He was a known multiple myeloma patient for 5 years.

**Figure 3:**
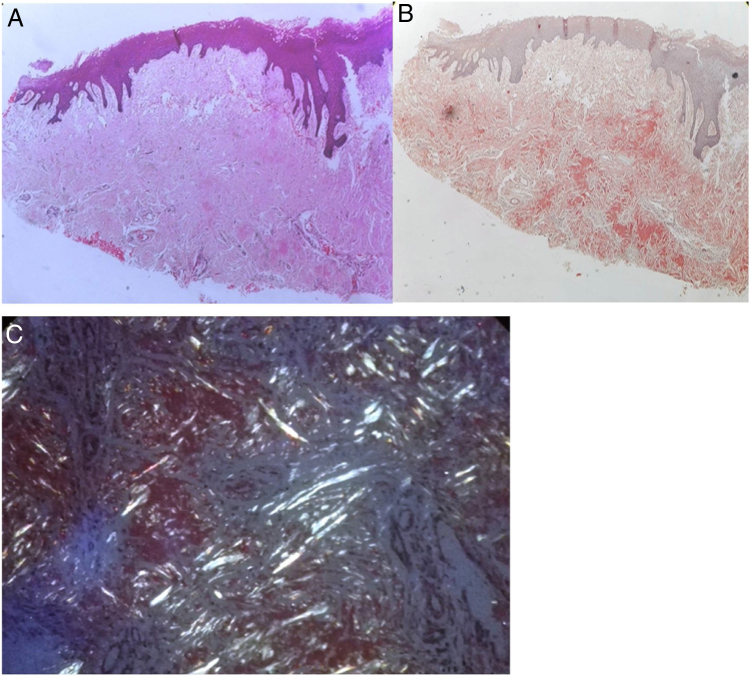
(A) Homogenous eosinophilic material in the corium (H&E, ×40). (B) Congo red stain with salmon pink positivity. (C) Green birefringence under polarized light.

An incisional biopsy was performed and revealed presence of amyloid just below the epithelium as well as in between muscles, which confirmed with Congo red stain and polarized light (Fig. 3a–c). The patient was symptomatically treated for oral discomfort and ulceration. In addition, upper gastrointestinal endoscopy was performed for further investigation for dysphagia.

Both patients gave consent for their inclusion in a research study.

## Discussion

Systemic amyloidosis results from the deposition of protein fibrils in various tissues and organs.^4^ Due to an alteration in the secondary structure of the proteins, the amyloid fibrils aggregate in the thermodynamically stable, insoluble form. Various types of amyloidosis can be identified by the specific biochemical composition of the protein subunits in question and may yield a range of clinical characteristics.^5^ The most common type is AL amyloidosis.

With congo red stain, amyloid deposits show a salmon pink color under ordinary light and shows a diagnostic apple green birefringence under polarized light. Ultrastructural examination shows non- branching fibrils with a diameter of 7.5–10 nm. The characteristic cross beta-pleated sheet conformation as demonstrated with X-Ray crystallography and infrared spectroscopy is the cause for the unique staining property, irrespective of the chemical composition of the fibrils.^6^

Amyloid deposit consists predominantly of fibril proteins. Normal proteins, facing some systemic conditions such as cell changes and chronic inflammatory disorders, might form insoluble fibrils that can result in organ damage and dysfunction.

Amyloidosis is a clinical entity with variable presentation, depending on the organs involved. In the oral cavity, amyloid deposits are rare. The head and neck region is affected between 12% and 90%, typically with involvement of the larynx and the tongue.^4^ Localized deposits of the larynx are common; however, involvement of the tongue is frequent because of the systemic disease.^5^ In the case described, multiple ulcerative lesions presented on the tongue and buccal mucosa. The most common head and neck presentations are hoarseness, nasal congestion, odynophagia, articulation problems, mandibular deformities, deglutition difficulties, airway obstruction, speech disorders and hypogeusia. Amyloidosis occurs more often in the sixth decade of life which is consistent with the case presented and show a slight male predominance. The male patient of this report had a history of slow progression of the lesions. The oral manifestations of amyloidosis are usually presented as nodules, papules, plaques and macroglossia and the colour of the mucosa may range from yellow, orange, red, blue and purple.^7^, ^8^ Yellow nodules or raised white lesions occurring predominately along the lateral border are also common. Amyloidosis in the tongue typically results in macroglossia, manifested by increased tongue volume, tongue protrusion beyond the alveolar ridge, speech impairment and dysphagia.^1^

There are currently 3 known forms of amyloidosis. The first, primary systemic amyloidosis, is a systemic condition with no known underlying cause. This differs from secondary systemic amyloidosis, which occurs with other underlying medical problems, such as tuberculosis and rheumatoid arthritis. This also includes patients with multiple myeloma, of which 10%–20% have associated amyloidosis,^2^, ^4^ similar to this report. Renal and cardiac diseases are seen in both primary and secondary systemic forms and are the most frequent causes of death. Other symptoms may include hypesthesias, syncope, macroglossia, and carpal tunnel syndrome. The third form of amyloidosis is localized, which occurs without any evidence of systemic involvement or underlying disease. It is rarer than the other forms, with the larynx being the most common site in the head and neck region. The disease can also present as periodontal disease, and lesions are exacerbated by the inflammation of the periodontium.^2^, ^4^

In many studies, oral conditions were the first detected signs of MM.^1^, ^3^ Some studies recounted that oral manifestations of MM were detected during the patient’s followup and the dental surgeons were able to participate in the diagnosis of the condition. These patients need to be followed by a multidisciplinary team who may improve the patient’s quality of life. Besides contributing to the diagnosis, dentists in the team need to take care of the dental health to prevent further complications, especially in patients who are candidates for anti-resorptive therapy.^6^

## Conclusion

This report cites the medical presentation that should help in expediting future diagnosis and recognition of oral manifestations of systemic amyloidosis as a sequence of systemic amyloidosis. MM develops in individuals aged 5thdecade to 8thdecade. Thus, it is significant that oral health care workers are aware of clinical and imaging changes suggestive of MM lesions in patients of susceptible age groups. Professionals should be encouraged to detect oral manifestations of MM in routine examinations early in order to facilitate a contribution for the increased survival and better prognosis.

## Conflicts of interest

The authors declare no conflicts of interest.

## References

[1] Reinish E.I., Raviv M., Srolovitz H., Gornitsky M. (1994). Tongue primary amyloidosis and multiple myeloma. Oral Surg Oral Med Oral Pathol..

[2] Talamo G., Farooq U., Zangari M., Liao J., Dolloff N.G., Loughran T.P. (2010). Beyond the CRAB symptoms: a study representing clinical manifestations of multiple myeloma. Clin Lymphoma Myeloma Leuk..

[3] Silva W.P.P., Wastner B.F., Bohn J.C., Jung J.E., Schussel J.L., Sassi L.M. (2015). Unusual presentation of oral amyloidosis. Contemp Clin Dent..

[4] Mollee P., Renaut P., Gottlieb D., Goodman H. (2014). How to diagnose amyloidosis of the tongue. Intern Med J..

[5] Aono J., Yamagata K., Yoshida H. (2009). Local amyloidosis in the hard palate: a case report. Oral Maxillofac Surg..

[6] Kolokotronis A., Stefanopoulos P., Xohellis M., Antoniades D. (2004). Oral manifestations of amyloidosis: report of two cases. J Oral Maxillofac Pathol..

[7] Elad S., Czerninski R., Fischman S., Keshet N., Drucker S., Davidovich T. (2010). Exceptional oral manifestations of amyloid light chain protein (AL) systemic amyloidosis. Amyloid..

[8] Sideras K., Gertz M.A. (2009). Amyloidosis. AdvClin Chem..

